# Efficacy of acetylsalicylic acid in schizophrenia: a literature review

**DOI:** 10.1192/j.eurpsy.2024.1508

**Published:** 2024-08-27

**Authors:** A. Aissa, S. Jedda, F. Askri, O. Maatouk, U. Ouali, Y. Zgueb, R. Jomli

**Affiliations:** ^1^Psychiatry A, Razi Hospital, Manouba; ^2^Psychiatry, Faculty of Medicine of Tunis, Tunis, Tunisia

## Abstract

**Introduction:**

There has been growing evidence to support the hypothesis that inflammation is involved in the pathogenesis of schizophrenia.

**Objectives:**

The aim of the present literature review was to assess the efficacy of acetylsalicylic acid (ASA) as an adjuvant agent in the treatment of an acute exacerbation of schizophrenia.

**Methods:**

We searched randomized clinical trials based on regular searches of MEDLINE, Embase, PubMed.

**Results:**

We included four studies. The results were in favor of the efficacy of ASA in the study where authors targeted early psychosis. Illness duration seems to predict response to anti-inflammatory agents.

**Conclusions:**

Further studies of early stages of schizophrenia are helpful.

**Disclosure of Interest:**

None Declared

